# Cellular Biomechanic Impairment in Cardiomyocytes Carrying the Progeria Mutation: An Atomic Force Microscopy Investigation

**DOI:** 10.1021/acs.langmuir.2c02623

**Published:** 2022-11-24

**Authors:** Brisa Peña, Shanshan Gao, Daniele Borin, Giorgia Del Favero, Mostafa Abdel-Hafiz, Nasim Farahzad, Paola Lorenzon, Gianfranco Sinagra, Matthew R. G. Taylor, Luisa Mestroni, Orfeo Sbaizero

**Affiliations:** † Cardiovascular Institute & Adult Medical Genetics, University of Colorado Denver Anschutz Medical Campus, Aurora, Colorado80045, United States; ‡ Bioengineering Department, University of Colorado Denver Anschutz Medical Campus, 12705 E. Montview Avenue, Suite 100, Aurora, Colorado80045, United States; § Department of Engineering and Architecture, University of Trieste, Trieste34127, Italy; ∥ Department of Food Chemistry and Toxicology, Faculty of Chemistry, University of Vienna, Währinger Straße 38-42, 1090Vienna, Austria; ⊥ Core Facility Multimodal Imaging, Faculty of Chemistry, University of Vienna, Wien, Währinger Straße 38-42, 1090Vienna, Austria; # Department F of Life Sciences, University of Trieste, Trieste34127, Italy; ¶ Polo Cardiologico, Azienda Sanitaria Universitaria Integrata di Trieste, Strada di Fiume 447, Trieste34127, Italy

## Abstract

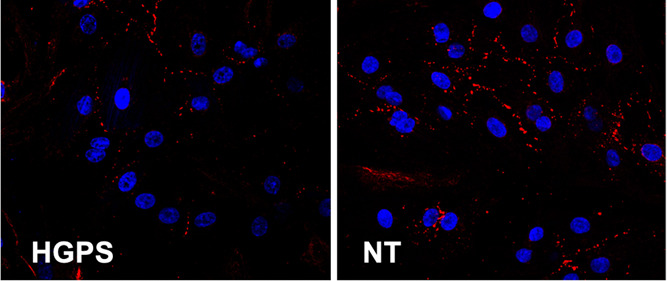

Given the clinical effect of progeria syndrome, understanding the cell mechanical behavior of this pathology could benefit the patient’s treatment. Progeria patients show a point mutation in the lamin A/C gene (LMNA), which could change the cell’s biomechanical properties. This paper reports a mechano-dynamic analysis of a progeria mutation (c.1824 C > T, p.Gly608Gly) in neonatal rat ventricular myocytes (NRVMs) using cell indentation by atomic force microscopy to measure alterations in beating force, frequency, and contractile amplitude of selected cells within cell clusters. Furthermore, we examined the beating rate variability using a time-domain method that produces a Poincaré plot because beat-to-beat changes can shed light on the causes of arrhythmias. Our data have been further related to our cell phenotype findings, using immunofluorescence and calcium transient analysis, showing that mutant NRVMs display changes in both beating force and frequency. These changes were associated with a decreased gap junction localization (Connexin 43) in the mutant NRVMs even in the presence of a stable cytoskeletal structure (microtubules and actin filaments) when compared with controls (wild type and non-treated cells). These data emphasize the kindred between nucleoskeleton (LMNA), cytoskeleton, and the sarcolemmal structures in NRVM with the progeria Gly608Gly mutation, prompting future mechanistic and therapeutic investigations.

## Introduction

The original description of Hutchinson–Gilford progeria syndrome (HGPS) was made in 1886 by Hutchinson. The name refers to a rare genetic condition in which symptoms simulating features of aging appear at a very young age (the word is derived from the Greek: “early” e v, “old, elderly”). Its prevalence is believed to be 1 in 18 to 21 million persons.^1^ A human A-type lamin gene mutation is what leads to HGPS (LMNA).^2,3^

Typically, HGPS patients show a single-base pair substitution (c.1824 C > T, p.Gly608Gly) within exon 11 of LMNA.^2,3^ A mutant protein called progerin is generated as a consequence of the mutation, which leads 150 nucleotides in lamin A to be deleted. Progerin maintains its C-terminal sequence but lacks a protein region that is mandatory for normal lamin A processing. Thus, progerin is stably farnesylated, a process that disrupts proper nuclear scaffolding.^4−9^ This truncated form of LMNA gathers in the nuclear envelope, sometimes causing alterations in the nuclear shape, and therefore alters several cell functions.^10,11^ The nuclear lamins are intermediate filament A-type (primarily lamin A/C) and B-type (lamin B1/B2) proteins, making a filamentous layer placed close to the inner nuclear membrane (INM). Even though lamins are directly implicated in the structural integrity of the nucleus, they have been found to intermingle with several proteins and play essential roles in chromatin organization, gene regulation, and signal transduction^12,13^ and have downstream effects on cell polarization, adhesion, and mechanics via cytoskeletal linker proteins.^14^ Lamins consist of an α-helical central rod domain edged by a short globular amino-terminal “head” domain and a longer carboxy-terminal “tail” domain.^15^ Several studies have already highlighted that cells carrying a progeria mutation have impaired mechanical properties.^8,16−19^ Nuclei of fibroblasts isolated from mice with Lmna knockdown and cells derived from HGPS patients are both mechanically weak,^20,21^ isolated nuclei assembled in lamin-depleted Xenopus egg extracts are highly fragile,^22^ and fibroblasts from patients have increased cellular sensitivity to mechanical strain.^12^ However, when dermal fibroblast cell lines from patients of different ages have been studied, Dahl et al.^21^ found that the mechanical properties of the lamina were altered in HGPS cells, but these changes did not lead to disease symptoms via increased nuclear fragility. They concluded that nuclear alterations may modify the mechanotransduction or the activity of mechanosensitive genes.^21^ In the heart of HGPS patients, premature aging of the vascular system causes atherosclerosis, myocardial infarction, and heart failure. In a recent gene-edited mice model of HGPS (HGPSrev), the mice appeared healthy at birth and, like humans, progressively developed HGPS symptoms, including vascular smooth muscle cell loss, vascular fibrosis, electrocardiographic anomalies, and precocious death. Remarkably, progerin suppression and lamin A restoration significantly improved the phenotype and increased the lifespan of this HGPS mice model.^23^ Although an altered cardiomyocyte structure has been described in laminopathies,^24−27^ they are less understood in progeria.

For these reasons, we used a multidisciplinary approach to study the mechanobiology of cardiomyocytes carrying the Gly608Gly LMNA mutation. We assessed mechanical properties, such as elasticity (Young’s modulus), adhesion work, and relaxation time, as well as contractile forces, beat frequencies, and durations, using the atomic force microscopy (AFM) technique, which has already been shown to be very useful for cellular research.^28−38^ These results were correlated with calcium transients’ analysis, immunostaining for gap junction proteins implicated in mechanotransduction in muscle contraction, and analysis of actin filaments and microtubules within the cellular cytoskeleton.

## Materials and Methods

### Isolation and Culture of NRVMS

NRVMs were prepared from six, 1–3 day old pups, as previously described, with minor modifications.^24,25^ The University of Colorado Denver Animal Care and Use Committee’s regulations were fulfilled for all animal studies. Using scissors, the ventricles and atria were separated, and the ventricles were then separated, dissociated in calcium-free and bicarbonate-free Hanks with Hepes (CBFHH) buffer containing Heparin (Sigma-Aldrich) (10 U·mL^–1^), and digested in a CBFHH solution containing 1.12 mg·mL^–1^ of trypsine (Gibco) and 20 μg·mL^–1^ of DNAse (Sigma-Aldrich). Two sequential pre-plating steps, on 100 mm dishes in Dulbecco’s modified Eagle’s medium (Gibco), were used to enrich cardiomyocytes (>90% purity) over non-myocytes. First, 4.5 g augmented with 5% bovine calf serum (Gibco) and then 2 mg mL^–1^ vitamin B12 (Sigma-Aldrich) and cultured unattached cells, predominantly myocytes, were harvested and cultivated in gelatin-coated dishes before being given various treatments and further examinations. A minimum of three separate cell isolations were used to test each experimental condition in triplicate.

### Isolation Adenoviral Constructs and Infection

Shuttle constructs were crafted in dual CCM plasmid DNA having GFP gene and human LMNA cDNA wild-type (WT) and mutant c.1824 C > T (p.Gly608Gly) (HGPS). Constructs were bicistronic, with the two inserts (LMNA and GFP) driven by two different CMV promoters to detect cells expressing LMNA protein using GFP as a marker of cellular infection. Constructs contained either human wild-type or human mutant LMNA genes. NRVMs were infected by adenoviruses at 50 multiplicities of infection in a serum-free medium, 6 h post-infection. The complete medium was replaced with cardiomyocytes, and the cells were incubated at 37 °C and 5% CO_2_. Tests were performed 48 post-infection since our data indicate that protein expression begins within 12–24 h post-infection and persists for at least 6 days, consistent with the time course of expression of exogenous molecules using the adenoviral system, as previously reported by several other groups.^24,39−42^ NRVMs not treated with the adenoviral construct (NT) were used as aditional controls and they were obtained from six, 1–3 day old pups. All the experiments were made from at least 3 independent experiments and from at least 3 cell isolations.

### Immunofluorescence

NRVMs were fixed in phosphate-buffered saline (PBS) containing 4% PFA for 15 min at room temperature. Cells were permeabilized with 1% Triton X-100 for 90 min, blocked with 2% bovine serum albumin (BSA) in PBS for 45 min, and left overnight at 4 °C with the following primary antibodies α-sarcomeric actinin 1:500 (ab9465, Abcam) and Connexin 43 1:500 (c6219, Sigma-Aldrich) in PBS with 2% BSA, at 4 °C. Then, the goat anti-mouse antibody conjugated to CY5 (A10524; Thermo Fisher Scientific) and the goat anti-rabbit antibody conjugated to Alexa Fluor 555 (A-21428; Thermo Fisher Scientific) were added, as secondary antibodies, at a dilution of 1:300 for 45 min at room temperature. DAPI (Life Technology) was then added (1:8000 in PBS for 2 min) for nuclei staining. For cytoskeleton assessment, Alexa Fluor 594 phalloidin (A12381; Thermo Fisher Scientific) was used at 1:500 dilution for 1 h in PBS with 2% BSA, at room temperature followed by DAPI staining as mentioned above. For microtubule staining, the primary antibody α-tubulin (2125s, Cell Signaling Technology) was used at a dilution of 1:200 with a goat anti-rabbit secondary antibody conjugated to Alexa Fluor 555 (A-21428; Thermo Fisher Scientific) followed by DAPI staining as mentioned above. Fluorescent images were taken from 4 regions of each sample (*n* = 3) from at least 3 independent cell isolations with a Zeiss LSM780 spectral, FLIM, 2P, SHG confocal. Within each experiment, instrument settings were kept constant.

### Immunofluorescence Analysis

The localization of Cx43 was performed using Matlab coding. Briefly, to calculate the amount of Cx43 localized between cells in percentage/area, a MATLAB script was written using MATLAB R2021A. The script used the RBG and YCbCr color spaces. Two different thresholds were applied to the red color channel and only pixel intensity values falling within that range were kept. Additionally, the Cr color channel in the YCbCr color space was used and only pixels falling within the 0–125 intensity range were kept. Calculated and reported on an Excel sheet was the percentage of isolated pixels. Actin filament thickness was analyzed manually using ImageJ. For both analyses, at least 10 confocal images were analyzed per group from 3 different independent experiments (pictures were taken using a 40× objective with an area of analysis of 11,293 μm^2^).

### Western Blot

Proteins were extracted from cells with RIPA buffer (Life Technology). Proteins were separated on polyacrylamide gels and transferred to poly(vinylidene difluoride) membranes (Millipore, Burlington, MA). Membranes were blocked with 5% nonfat milk in TBS-T (Tris-buffered saline, 0.1% Tween 20) at room temperature for 1 h. To detect protein expression, antibodies specific for Connexin-43 (c6219, Sigma-Aldrich), Progerin (Sigma-Aldrich, St. Louis, MO), and GAPDH (AM4300; Invitrogen) were used. Membranes were subsequently incubated with horseradish peroxidase-conjugated secondary antibody, and signals were created using boosted chemiluminescence substrate (Thermo Fisher Scientific).

### AFM—Cell Indentation

An AFM (JPK NanoWizard 4a with CellHesion technology) was used to acquire force–displacement curves for the cardiomyocytes previously described. By carefully following a standard operating procedure, samples were processed to prevent measurement bias brought on by cell heterogeneity. This comprises (1) cells made in accordance with a specific protocol for every cell type. Additionally, AFM experiments were always carried out on the same day that biochemical (expressions) analyses were collected, (2) multiple measurements from various cells were gathered to account for variability and determine the “average” data, and (3) cells were examined and their morphological details were noted (an optical light microscope was used for cell selection throughout the tests). A standard cantilever holder cell for operating in liquid at a controlled temperature was used in the AFM setup. A polystyrene microsphere (diameter of ∼7 μm) coated with a layer of gold was used as the AFM tip, to precisely apply a compression force “normal” to the nucleus. AFM probes were cleaned, by inserting them in Tween (2% for 30 min), to remove contaminant molecules adsorbed on the probe surface. All tests were carried out on living, undamaged cells in a cell culture medium. Only well-spread cells with a well-defined morphology were investigated. Large cell clusters (more than 6 cells) were excluded from the analysis as well as cells with a round shape and a dark edge (dying cells). The relationship between the displacement of the cantilever and the cell indentation was obtained from the force curves. Penetration depth was calculated by comparing the curve recorded on the glass substrate with that recorded in the cell. To calibrate the cantilever displacement signal, force *versus* displacement curves were recorded on the rigid substrate (glass) of the cell. The AFM tip was moved toward the cell at speeds of 1 μm/s. The speed range was chosen to avoid cell movement (at a low compression speed) or hydrodynamic force contribution (significant at a high speed).^43^ The distal regions, away from the nucleus, were avoided since measurements performed around the nucleus were less affected by artifacts due to substrate stiffness. All experiments were performed at the same velocity since these AFM tests were rate dependent. We evaluated nuclear elasticity using at least 40 cells for each condition (NT, WT, and HGPS mutated NRVMs), and the same operators performed the experiments. These data were sufficient to establish a statistically significant difference. The test duration was never longer than 45–50 min to protect cell viability.

### Modeling Cells Stiffness

Cell stiffness is described as elastic modulus or Young’s modulus, acquired using the AFM and fitting the curve of force *versus* indentation. Pushing the AFM tip into the cell nuclear region provides the “global stiffness” because it reflects the whole joint stiffness of the structures inside the cell. From the time when the earliest AFM studies of soft biological samples were carried out,^44,45^ the dominant method of evaluating AFM indentation data to gauge elasticity has been the so-called “Hertz-Sneddon model” of contact between two elastic bodies. The Hertz model offers an approximation of the elasticity, and it is clear that in any case it will be afflicted by an error since it involves the assumption that contact surfaces are uninterrupted and frictionless and that their deformations are irrelevant. Although these assumptions are not an exact match for real cells due to the heterogeneous cell structure, the Hertzian model is useful for obtaining a description of cell elasticity. However, elasticity values calculated using various models differ from each other, suggesting a great influence on the process used.^46,47^ In fact, in the literature, data regarding the elasticity of cellular nuclei are quite divergent, with values ranging from few Pascal to several kilo-Pascal, differences which are likely due to a range of factors such as cell type, measurement techniques and conditions, interpretation models, and the influence of the cell support visible even at small cell indentation depths.^48−50^ The present study uses the same protocol/methodology and the same model for all cells and indentations. Therefore, even if we do not think that we can provide absolute stiffness values, the data can allow us a valid comparison between the different cell lines within the presented experiments. In the present work, we used the Hertz–Sneddon model for spherical tips.^51^
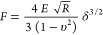
1where *F* is the load force, *E* is the Young’s modulus, ν is the Poisson ratio, and δ is the probe penetration into the cell. Poisson’s ratio was presumed to be 0.5. Finally, cardiomyocytes exhibiting contractile activity (<15% of total cell number) were not included in the analysis due to difficulties in establishing a reliable baseline.

### Adhesion Work

Adhesion work in this case is meant as the process of detachment of the cell membrane from the AFM sphere. During the AFM tip retraction to the maximum cell height (unload curve), the AFM sphere adheres to the cell membrane, causing a reverse probe deflection below the baseline. The adhesion work (or de-adhesion work) was evaluated by integrating the area below baseline, until the final force interaction that resulted in detachment of the cell membrane from the globular tip.

### Cell Relaxation Time

**Figure 1:**
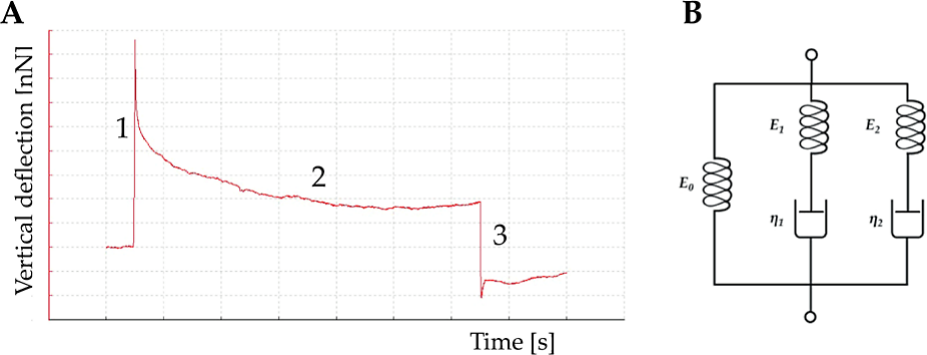
Cartoon illustrating a cell relaxation time test. (A) Curve obtained from the AFM tip movement *vs* time; (B) generalized Maxwell model with one spring in parallel with two Maxwell elements used to obtain relaxation time data from the curve shown in (A).

AFM force–deformation curves were also used to evaluate cell viscoelastic behavior. If the cell response is elastic, the indentation and retraction curves should be indistinguishable. However, typically, there is a substantial difference between the indentation and retraction curves. This hysteresis indicates that the response is not entirely elastic. The cell relaxation time was evaluated in a complementary way since the AFM also allows monitoring of the time progress of the cantilever’s force and the cantilever’s vertical position at all stages. These tests were carried out by acquiring force-relaxation responses in the so-called constant height mode Figure 1A.^52^

The figure illustrates a characteristic time course of deflection signals during a test. Succinctly, the AFM tip approaches the cell surface with a speed of 1 μm/s (zone 1 in Figure 1A). The indentation depth for each cell is set to 20% of its initial height, and the movement of the piezo scanner in the *Z* direction is adjusted with the deflection of the cantilever during the cell compression. The locus where the force sharply increased acts as the contact point between the tip and the surface. Subsequently, the cantilever base position is held constant for a fixed time, while the cantilever’s force changes and is recorded with time (zone 2 in Figure 1A). Figure 1 illustrates that the loading force decays with time. This behavior is ascribed to the cell’s viscoelastic properties. After the loading time, the AFM tip is withdrawn (zone 3 in Figure 1A). For these experiments, a dwell period of 60 s was used to minimize the drift. The stress relaxation measurements were performed only one time per cell. During the test, we assumed an approximately constant strain on the cells, while the force was recorded over time. This is due to the fact that, in comparison to the overall indentation, the change in cantilever deflection during relaxation was insignificant. The stress relaxation curves were first shifted along the *y*-axis to move the baseline (minimum) force to zero. The curves were then normalized by setting the maximum force value to 1, meaning that all the relaxation data fell from 0 (minimum of the baseline) to 1 normalized force range. Force data acquired during the dwell time (the relaxation phase) were divided by the tip-cell contact area, which was assumed to be constant during dwell time and represented by the projection of the spherical cap of the AFM probe in contact with the cell after the indentation. The obtained stress data were divided by deformation (i.e., indentation/initial cell height) to obtain the relaxation modulus *E*(*t*). Experimental data were fitted with a double-exponential eq 2, consistent with a generalized Maxwell model with one spring parallel with two Maxwell elements (Figure 1B). This model was preferred over single- and tri-exponential equations since the first did not describe well the first part of the relaxation curve, whereas the latter did not significantly enhance the quality of fitting.
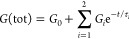
2

In eq 2, *G*(tot) is the normalized force profile and τ_1_ and τ_2_ are the two characteristic relaxation times. *G*_0_ corresponds to the Maxwell spring element and accounts for the equilibrium modulus; that is the asymptotic value of *G*(tot) for *t* → ∞. *G*_*i*_ and τ_*i*_ are the normalized force and relaxation time of the *i*-th Maxwell element, respectively. Considering Figure 1B, τ_*i*_ is equal to η_*i*_/*G*_*i*_, where η_*i*_ is the viscosity of the *i*-th Maxwell element. Specifically, τ_1_ governs the short-term relaxation behavior, whereas τ_2_ corresponds to the slow relaxation time. The normalized curve was also used to calculate the percent relaxation at the end of the applied stress
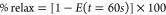
3

### Beating Properties

Beating properties were also assessed using AFM. When making the beating recordings, some issues should be considered, like the size of the cell, but mostly if they are isolated cells or small clusters. There are already some works in which the beating behavior of single cells or cluster were taken into consideration.^53−56^ In all of them, the two distinctive conditions had somewhat different behavior. For example, Sakamoto et al.^56^ used the coefficient of variability (CV) to assess the relationship between the fluctuation of the interval of spontaneous beating (IBI) and found that isolated cells had both a shorter IBI and a widely dispersed mean IBI distribution compared to the beating cluster. Indeed, first, we tested isolated beating cells as well as small clusters to consider variation in beating behavior. However, we observed that remote cells had great mechano-dynamic variability at some point during the acquisition time. Instead, the central cell of small clusters constantly confirmed a greater reproducible beating pattern. Therefore, for testing cell elasticity, cell adhesion, and cell relaxation, we tested single cells; however, for beating analysis, we reported data obtained from the central cell of small clusters (formed by 3–6 cells: well-spread and well-defined cells with neighboring cells). Cells were gently touched by the cantilever sphere using 2 nN of force. The cantilever tip was kept in position for a minute interval, while deflection data were collected at an acquisition rate of 2 kHz. Data on deflection was multiplied by the spring constant to convert it to force. The resulting data were analyzed using the MATLAB homemade software to calculate the force, frequency, duration (peaks distance), and full width at half-maximum of each beat. The Poincaré plot was used to assess the beating rate variability (BRV), as recommended by Tulppo et al.^57^ for analyzing heart rate signals.

The Poincaré plot helps to assess components of the beat variability related to short- and long-term connections of the signal.^58,59^ It is a scatter graph of the peak’s interval plotted against the preceding peak’s interval. The first peak interval [*i*] represents the *x*-coordinate; the second interval [*i* + 1] represents the *y*-coordinate. By fitting an ellipse with its center coincident with the centroid of the ellipse, the quantitative analysis of the plot is realized.

**Figure 2:**
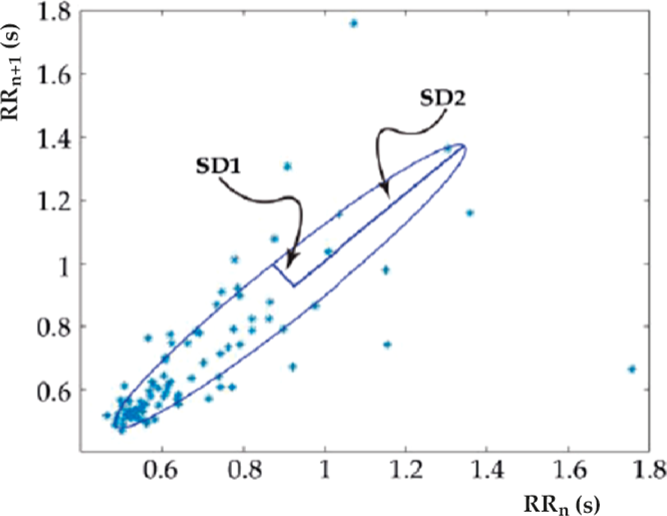
Typical Poincaré plot. The abscissa represents the RR interval of the current beat and the ordinate represents the RR interval of the succeeding beat. The data points are fitted with an ellipse.

The cumulative mean of the intervals is represented by the intersection of both ellipse axes. The minor and major axes of the ellipse have lengths of 2SD1 and 2SD2, respectively, where SD1 and SD2 are the dispersions along the main axis and perpendicular to the minor axis, as described by ref.^57^ While SD2 is the standard deviation of the long-term interval variability, SD1 is the standard deviation of the instantaneous (short-term) beat-to-beat variability. Another index derived from this plot is the axes ratio *R* = SD1/SD2 measuring the balance between long- and short-term beating variability. Figure 2 is an example of a Poincaré plot.

### Calcium Measurements

Cytosolic-free calcium ([Ca^2+^]_*i*_) was monitored in single cardiomyocytes by using the fluorescent calcium probe Fura2-AM as described by Huang et al.,^60^ with minor modifications.^61^

[Ca^2+^]_*i*_ measurements were performed on a digital fluorescence-imaging microscopy system built around a Zeiss Axiovert 135 (Oberkochen, Germany) as previously described.^62^ A modified dual-wavelength microfluorimeter (CAM-230; Jasco Intl. Co., Ltd., Tokyo, Japan) supplied the 340 and 380 nm excitation light, and a low light level CCD camera captured the fluorescence images (Hamamatsu Photonics, Hamamatsu, Japan). The camera output was fed into a digital image processor where video frames were digitized and integrated in real time. Two frames per second were used for the image acquisition. Determination of ratio and [Ca^2+^]_*i*_ calculation was performed pixel by pixel on pairs of corresponding 340 and 380 images according to Grynkiewicz et al.^63^*R*_max_ and *R*_min_ were assessed using ionomycin (40 μM) with Ca^2+^ (30 mM) or a stoichiometric excess of EGTA, respectively. Hence, *R*_max_ represented the ratio of 340 nm/380 nm signals under saturating conditions of calcium, while *R*_min_ represented the same ratio in the absence of Ca^2+^. Values used for calibration included *R*_max_ = 650, *R*_min_ = 200, *K*_d_ = 224 nM, and *f*_380min/f380max_ (ratio of fluorescence at high Ca^2+^ to that at low Ca^2+^) = 2. Time-based plots were calculated from ratio images, and each plot shows the mean value of the variation in the fluorescent signal at the single cell level. In order to prevent compartmentalization of the dye, cell loading was carried out at room temperature. To minimize spontaneous [Ca^2+^]_*i*_ spiking by cardiomyocytes during the test, cells were maintained at 37 °C for 30 min before starting [Ca^2+^]_*i*_ recordings. The homogeneous distribution of the fluorescent dye was carefully checked before starting [Ca^2+^]_*i*_ recordings.

### Statistics

Experimental data were analyzed using the GraphPad Prism software. All data were first subjected to the D’Agostino–Pearson normality test, and then ordinary one-way ANOVA and Holm–Sidak’s multiple comparison tests for normal distributions or the Kruskal–Wallis test with a Dunn’s multiple comparison test otherwise, both with a two-tailed *P-*value, were used. Data in the text are reported as mean (normal distribution) or median (non-normal distribution) values ± standard error.

## Results and Discussion

For the biomechanical analysis, isolated beating cells and individual cells with neighboring cells were tested to assess if dissimilarities in beating behavior were present. Noticeably, cells among neighboring cells (3–6 cells) always showed a more regular and stable beating pattern than lonely cells without neighboring cells, and consequently, all further data in this study are related to individual cells with neighboring cells obtained from cells seeded at a density of 20000 cells/well. A series of experiments on abnormally shaped nuclei have also been carried out. The typical trend was that HGPS nuclei were smaller and sometimes characterized by small blebs. However, even though there is a trend in having a smaller degree of circularity, this difference is not statistically relevant.

### Biomechanical Properties

**Figure 3:**
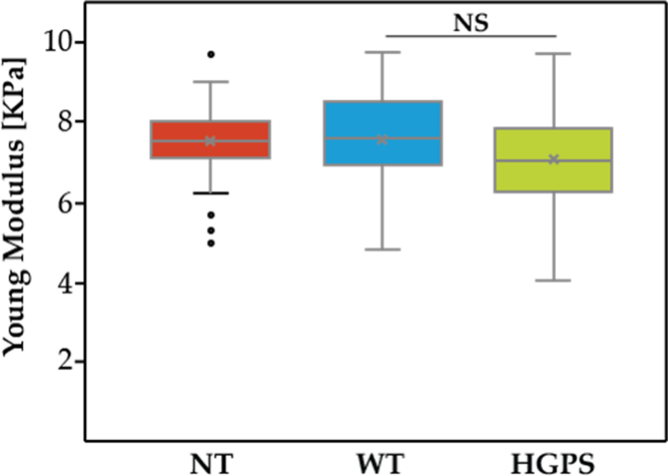
Cell elasticity expressed as Young’s modulus assessed by cell indentation (NT-*n* = 55 tested cells, WT-*n* = 61, HGPS-*n* = 61).

The cell’s elasticity expressed as Young’s modulus (*E*) is shown in Figure 3. The trend in data indicates that cells carrying the mutation (HGPS = 7.20 ± 1.1) might be less stiff than both control (NT = 7.52 ± 0.82) and wild type (WT = 7.56 ± 1.1); however, this slight decrease in the elastic moduli is not statistically significant (NT *vs* WT *p* = 0.802, NT *vs* HGPS *p* = 0.054, WT *vs* HGPS *p* = 0.051).

**Figure 4:**
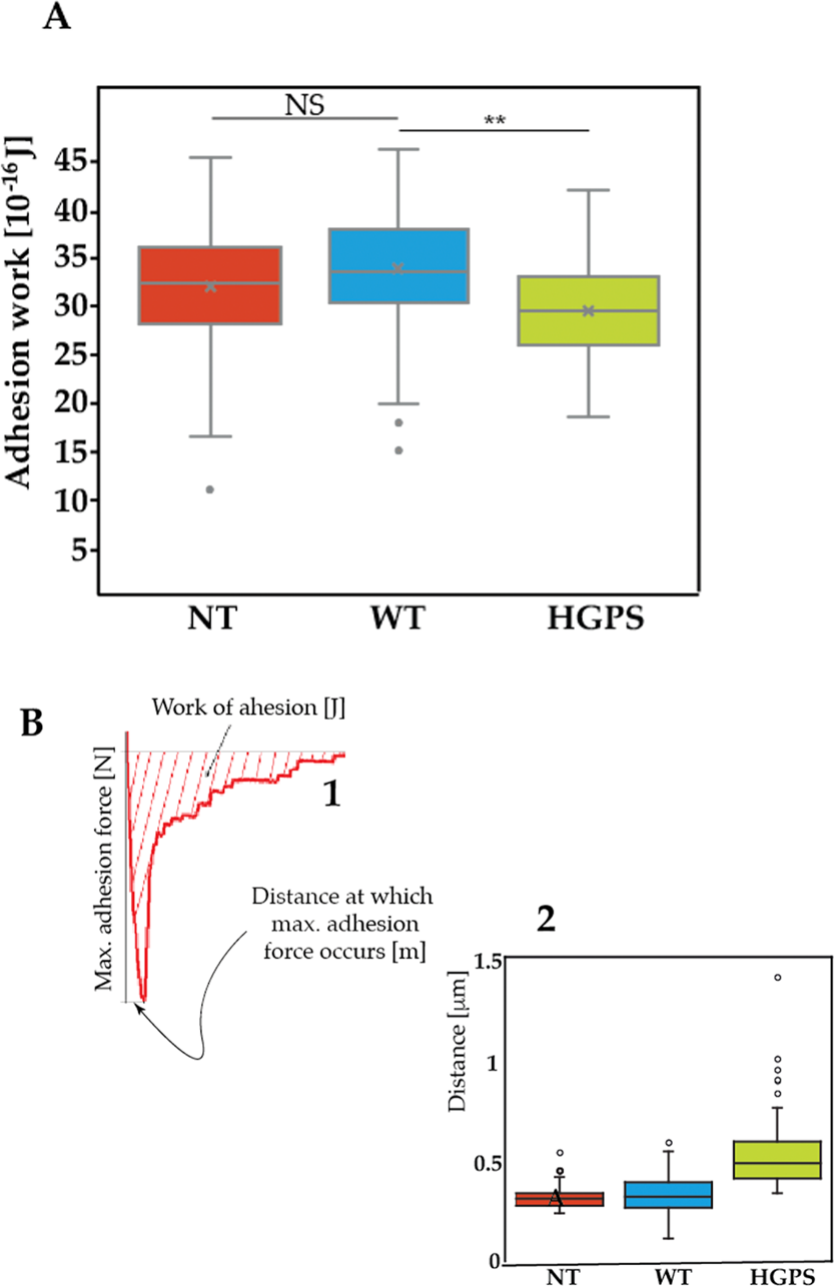
(A) Cell adhesion work is calculated by integrating the area obtained during a cell indentation test until the cell membrane detachment from the spherical tip (**p = < 0.001). (NT-*n* = 59 tested cells, WT-*n* = 61, HGPS-*n* = 61). (B) (1) Cartoon showing an enlargement of the area of the AFM curve recorded during the “unloading” part of the cell indentation test. (2) Data showing the position of the distance at which maximum adhesion force occurs (NT-*n* = 55 tested cells, WT-*n* = 61, HGPS-*n* = 61).

For the heart to work as an electromechanical syncytium, cardiac myocyte adhesion is crucial. Therefore, cell adhesion is a key biological property for maintaining multicellular structures. Adhesion’s mechanical function is to supply the energy (adhesion energy) necessary for cells to adhere to their surroundings in the best possible way. Ionic and hydrogen bonds that are created between cells and their adhesion partners are what produce the adhesion energy. In our AFM tests, by integrating the area obtained until the cell membrane detachment from the spherical tip, we found that the cells carrying the mutation (HGPS = 29.49 ± 4.9) show a reduced work of adhesion (NT = 32.1 ± 6.1) (WT = 33.83 ± 6.32) (NT–WT *p* = 0.0541, NT–HGPS *p* = 0.00432, WT–HGPS *p* = 0.00256); see Figure 4A. Additionally, on the same retracting curve, it is possible to identify the distance at which the maximum force of adhesion occurs (see Figure 4B-1). In this case, the distance for the HGPS cells is further along the retracting curve (see Figure 4B-2), substantiating that the adhesion work is reduced as well as that the slope of the AFM curve is lower, which confirms that there is a trend toward a lower stiffness.

**Figure 5:**
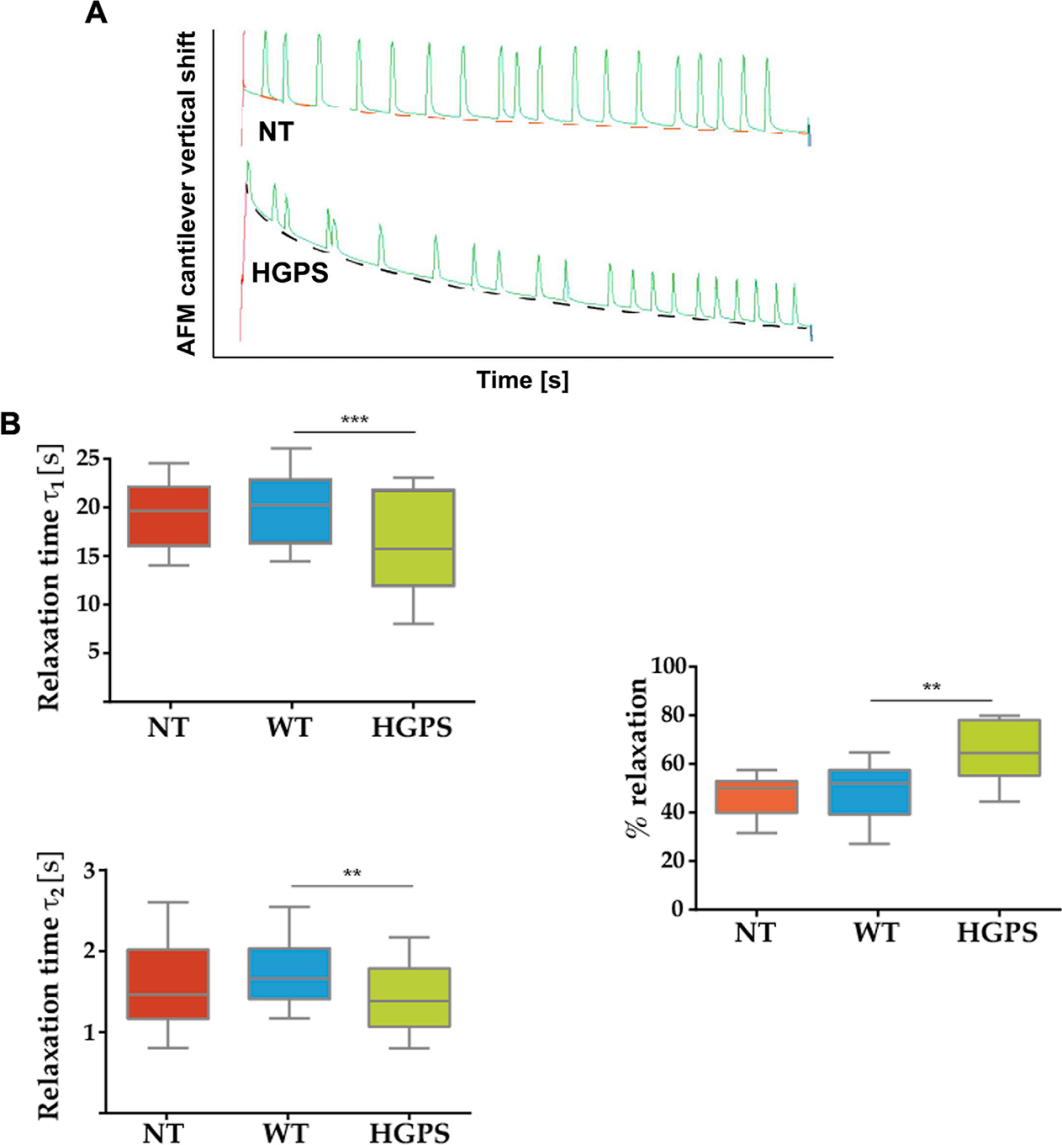
(A) Typical curves were recorded during a stress relaxation experiment for control (NT) and progeria (HGPS) cells. Dotted curves are those used in the generalized Maxwell model. (B) Data obtained from the generalized Maxwell model used (***P < 0.0001, **P < 0.001) (NT-*n* = 30 tested cells, WT-*n* = 30, HGPS-*n* = 31).

Since the mean values of the elasticity data (Young’s modulus) showed a visual trend for the HGPS cells (although not statistically different), indicating that they might be slightly softer compared to the NT control and wild type, we further investigate cell stiffness performing stress relaxation experiments. By fitting the stress–relaxation curves (Figure 5A), the cellular relaxation times obtained at τ_1_ and τ_2_ highlighted a decreased relaxation time in HGPS cells (τ_1_: 16.15 ± 9.8 s for HGPS *vs* 19.03 ± 6.9 s for NT, *p* = 0.0016 and 21.11 ± 7.4 s for WT, *p* = 0.00011 respectively; τ_2_: 1.16 ± 0.34 s for HGPS *vs* 1.40 ± 0.57 s and 1.62 ± 0.37 s for NT and WT, *p* = 0.044, *p* = 0.0018, respectively). Indeed, HGPS cells relaxed more than control cells: 62.12 ± 10.91% for HGPS *versus* 51.47 ± 6.44% for NT, *p* = 0.005, and 53.22 ± 7.25% for WT, *p* = 0.008 (Figure 5B).

Our findings indicate that there is a modification in the cell adhesion properties and cytoskeletal biomechanics in the HGPS NRVM model.

### Beating Properties

**Figure 6:**
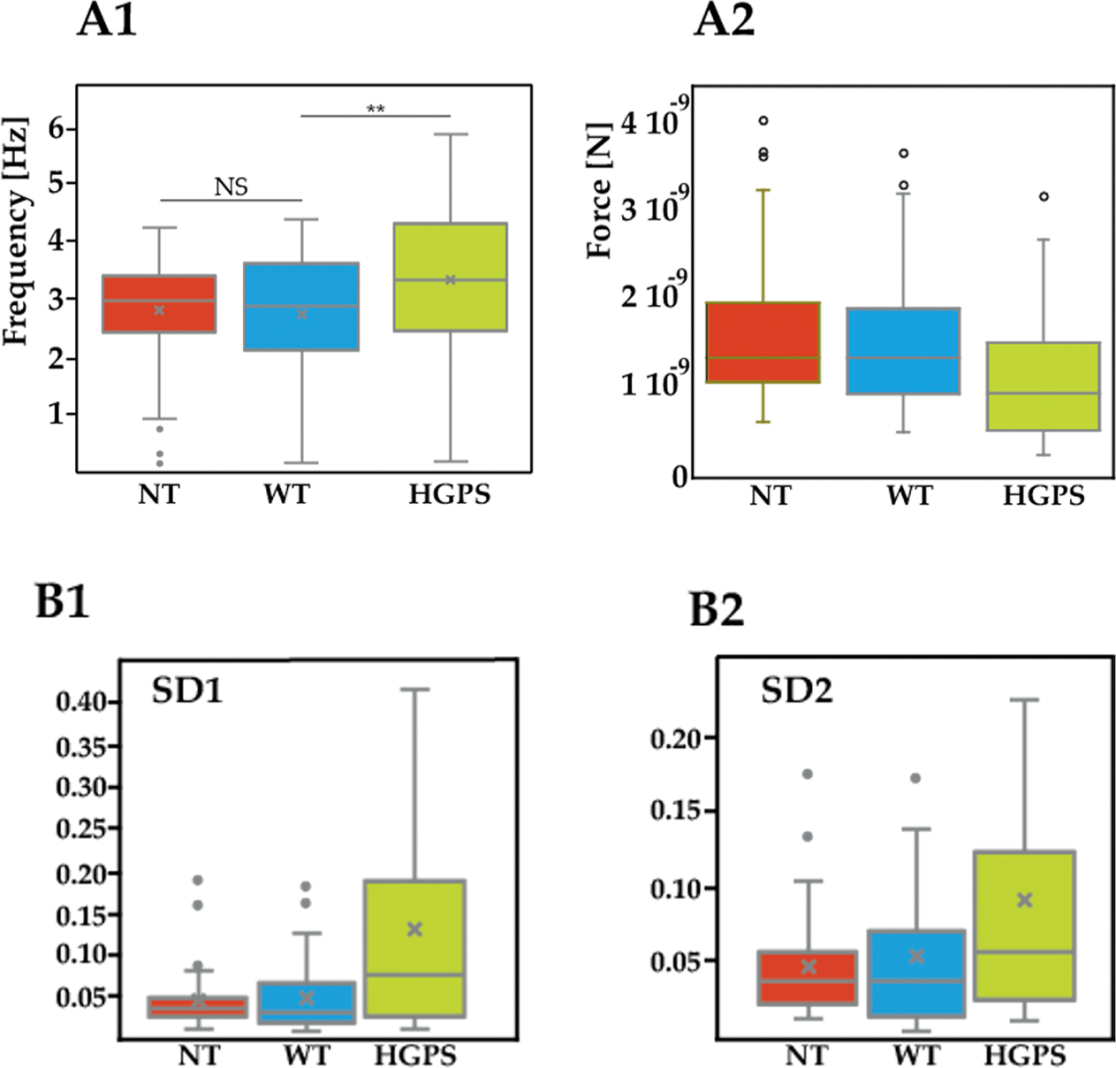
Beating data for the studied cells. (A1) Beating frequency; (A2) beating force. (B) Data from the cell beating behavior and analyzed with Poincaré theory. (B1) SD1 is the standard deviation of the instantaneous (short-term) beat-to-beat variability, while (B2) SD2 is the standard deviation of the long-term interval variability (NT-*n* = 55 tested cells, WT-*n* = 61, HGPS-*n* = 61).

As far as beating force is concerned, NT and WT cardiomyocytes do not show any statistical difference between them. However, HGPS cells showed a higher beating frequency and a reduced force (Figure 6A1–A2) compared to controls. Furthermore, as far as the BRV is concerned, Poincaré plots (Figure 6B1–B2) displayed a statistical difference for the HGPS cells: in fact, both SD1 (NT = 0.14, WT = 0.18, HGPS = 0.73 ms) and SD2 (NT = 0.24, WT = 0.24, HGPS = 0.82 ms) were increased, suggesting an irregular, arrhythmic beat.

### [Ca^2+^]_*i*_ Transients

**Figure 7:**
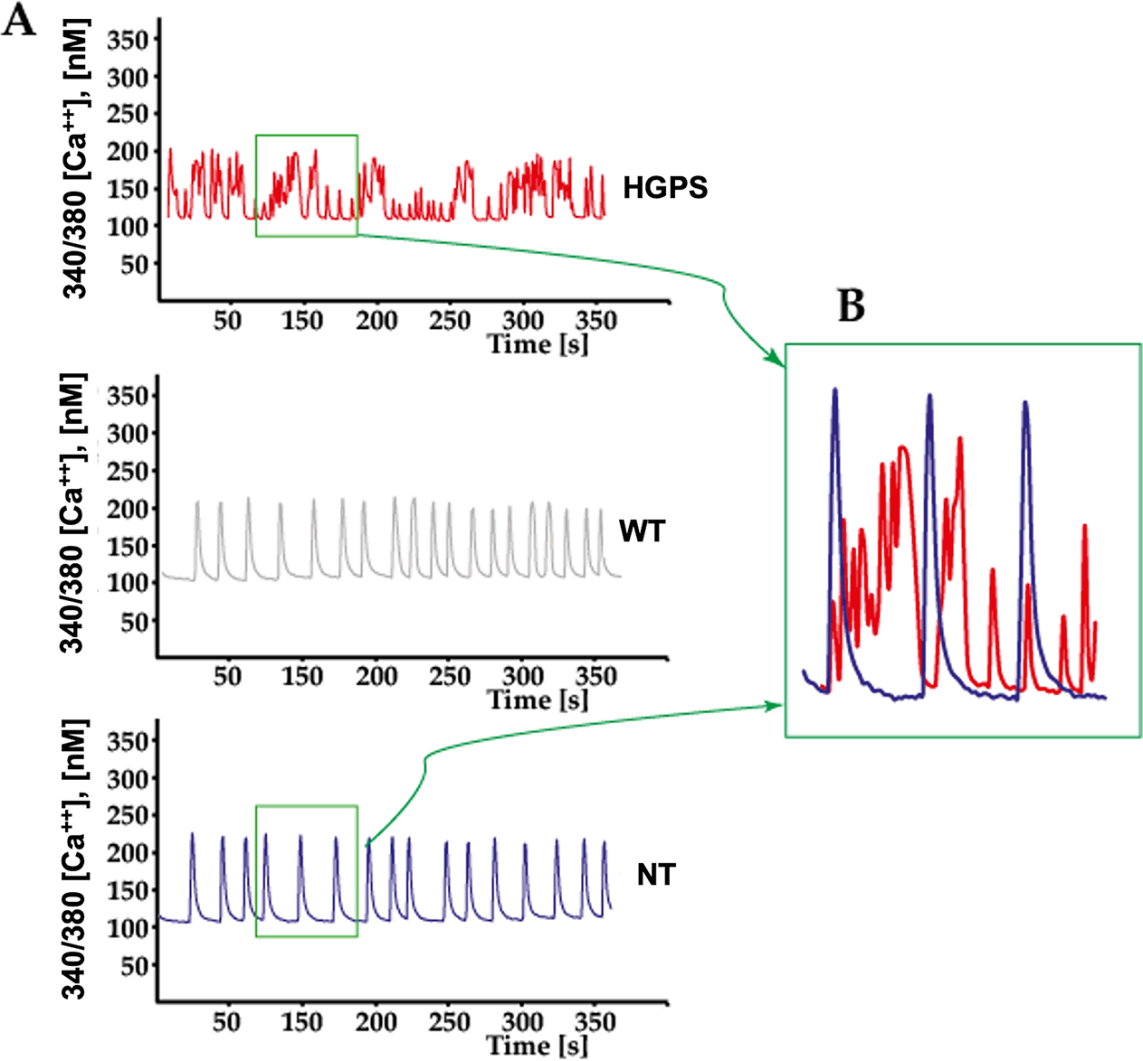
(A) Representative traces of [Ca^2+^]_*i*_ transients acquired during Ca^2+^ imaging recordings. (B) Overlay of the selected parts of traces from control and HGPS [Ca^2+^]_*i*_ transients (NT-*n* = 30 tested cells, WT-*n* = 30, HGPS-*n* = 30).

Ca^2+^ imaging was used to correlate the mechano-dynamic behavior previously assessed with AFM. Using the Fura-2 indicator, the amplitude and kinetics of intracellular [Ca^2+^]_*i*_ transients in cardiomyocytes (Figure 7) showed an arrhythmic profile in HGPS cells when compared with NT and WT cardiomyocytes (Figure 7A,B), suggesting a dysfunctional beating activity.

### Altered Connexin 43 (Cx43) Localization in Mutant HGPS NRVMs

**Figure 8:**
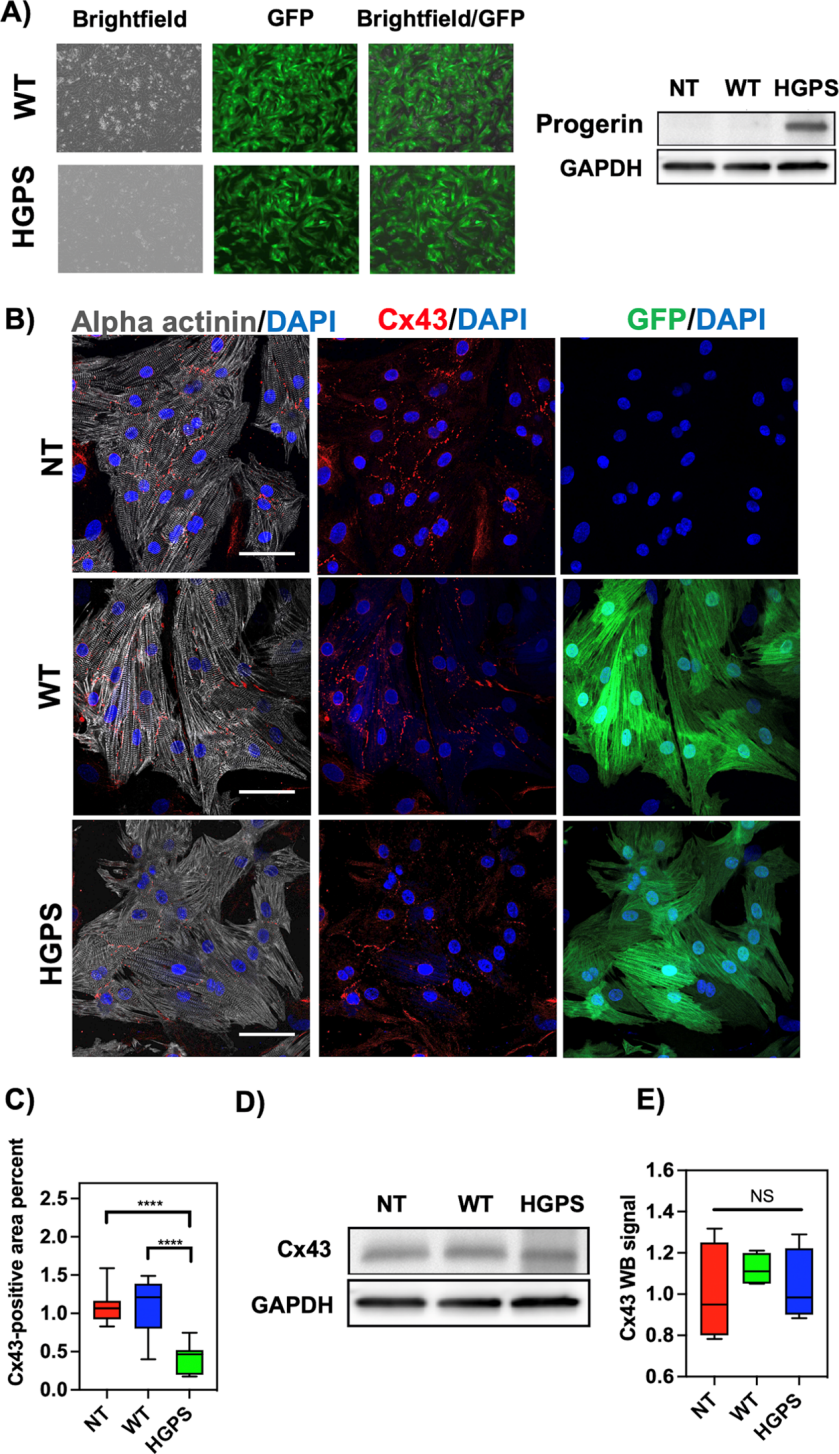
(A) Progerin expression in cardiomyocytes after adenoviral infection was confirmed by GFP viral fluorescence and by western blotting. (B) Fluorescence images of sarcomeric α-actinin (gray), connexin 43 (red dots), LMNA GFP (green), and DAPI (blue) staining of NRVMs: From top to bottom: non-treated NRVMs, wild-type-infected NRMVs and HGPS-infected NRVMs. (C) Quantification of Cx43 gap junction localization area percent: significant differences in Cx43 gap-junction area percent were observed between HGPS-infected cells *vs* the non-treatment cells and WT-infected cells. Data show the minimal and maximum values (*n* = 5). HGPS *vs* non-treated cells: (****P < 0.0001; HGPS *vs* WT: ****P < 0.0001). No significant differences were observed between the WT-infected cells and the non-treated cells. Scale bar: 100 μm. (D,E) Quantification of global Cx43 by Western blot: no significant differences were observed between groups.

To confirm the expression of progerin in the NRVM after adenoviral infection and the efficacy of the transfection, fluorescence images were taken and western blotting was performed as shown in Figure 8A. Figure 8A shows representative images of NRVM positive GFP-adenoviral infection and progerin expression in NRVMs after infection by western blotting, which corroborates that cells overexpress progerin. We further analyzed the localization and expression of Cx43, which is a member of the gap junction family, and a protein accountable for direct cell-to-cell communication between neighboring cells. Numerous investigations have established that Cx43 localization is essential for cardiac cell impulse propagation and thus normal cardiac function in the healthy human heart.^64,65^ Therefore, variations in the localization of Cx43 can be a prominent mechanism for arrhythmias.^66^ Since both AFM results and calcium transient highlighted an arrhythmic profile of HGPS NRVM, we investigated the levels and localizations of the gap junctions through immunostaining for Cx43. Figure 8B shows characteristic images of NRVM stained for the cardiac marker, α-actinin (gray), the gap junction marker Cx43 (red), the GFP virus (green), and the cell nuclei marker DAPI (blue). As shown in Figure 8C, HGPS cells presented a significant decrease of Cx43-positive localization area percent (0.41% ± 0.17; *p* = 0.001) when compared with the NT control (1.08% ± 0.2) and the WT control (1.11% ± 0.35). No substantial variances were found between the NT cells and the WT cells. However, no significant difference between the groups was observed regarding the global amount of Cx43 as shown by the western blot (Figure 8D,E). The results suggest that a decrease in Cx43 localization may be responsible for the mechanisms triggering arrhythmias in the HGPS NRVM.

### Cytoskeletal Structure: Analysis of Actin Filaments and Microtubules

**Figure 9:**
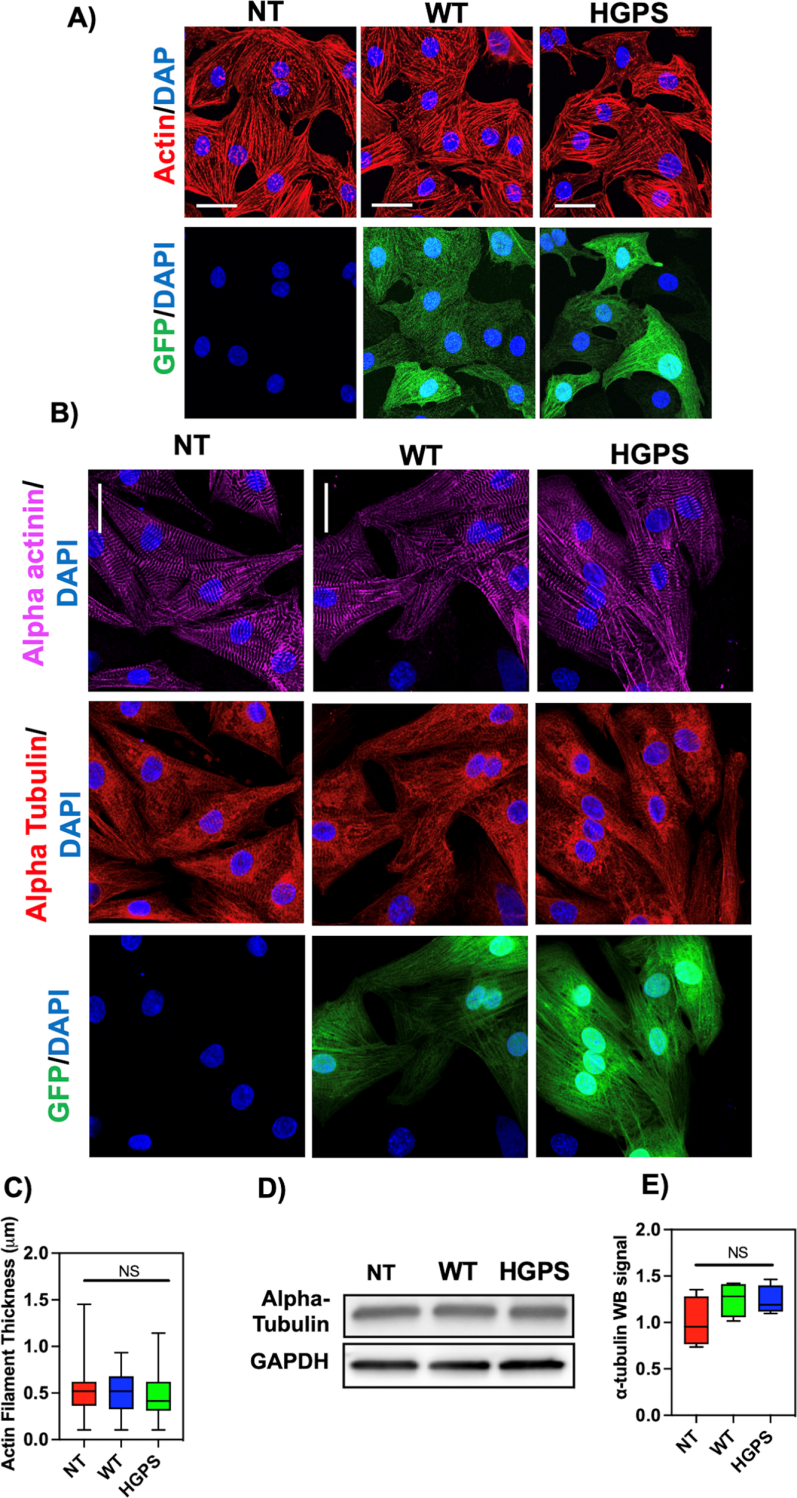
Cytoskeleton analysis: (A) fluorescence images of phalloidin-actin (red), LMNA GFP (green), and DAPI (blue) staining of NRVMs: from left to right: non-treated NRVMs, wild-type-infected NRMVs, and HGPS-infected NRVMs. Scale bar 25 μm. (B) Fluorescence images of α-actinin (pink), α-tubulin (red), the GFP virus (green), and DAPI (blue) staining of NRVMs: from top to bottom: non-treated NRVMs, wild-type-infected NRMVs, and HGPS-infected NRVMs. No significant morphological differences were observed between the groups. Scale bar 25 μm. (C) Quantification of the actin filament thickness: no significant differences were observed between the groups. (D,E) Quantification of alpha-tubulin by Western blot: no significant differences were observed between the groups.

Actin filaments are one of the main types of cytoskeletal polymers that control the shape and mechanics of cells. They enable the development of isotropic networks, bundled networks, and branched networks, among other highly ordered, stiff structures. Among other polymers in the cytoskeletal, actin filaments provide mechanical structure to the cell.^67^ Therefore, to address the possible involvement of actin filaments in the altered HGPS biomechanics, we analyzed the actin filament (Figure 9A) and microtubules (Figure 9B) of HGPS NRVM regarding their morphology and thickness and compared them with NT control and WT NRVMs. Figure 9A shows representative images of NRVM stained for the filament actin marker phalloidin (red), the GFP virus (green), and the cell nuclei marker DAPI (blue). As shown in Figure 9A, there are no morphological differences between the groups regarding actin filaments. Although HGPS cells present a lower value for actin filament thickness (0.49 μ ± 0.25) when compared with the NT control (0.52 μ± 0.23) and the WT control (51 μ ± 0.22), the results were not significantly different (Figure 9C). Furthermore, we analyzed the microtubules of HGPS NRVM regarding their morphology. Similar to actin filaments, microtubules are cytoskeletal polymers of tubulin that provide a cellular structural shape. Figure 9B illustrates representative images of NRVM stained for α-actinin (pink), α-tubulin (red), the GFP virus (green), and the cell nuclei marker DAPI (blue). We did not observe any significant morphological differences between the groups, and there were no significant differences between groups in the amount of tubulin as assessed by Western blot (Figure 9D,E). These results correlate with our findings mentioned above regarding the Young’s modulus of the cells in which no significant differences were found between the samples.

HGPS is defined as a laminopathy, being part of a family of diseases caused by mutations in the lamin A/C gene responsible for the proteins that make up the nuclear lamina. The nuclear lamina is composed of intermediate filament A-type (primarily lamin A/C) and B-type (lamin B1/B2) proteins. The nuclear lamina has a critical role in maintaining a nuclear architecture and stability as well as chromatin organization and function. Enormous progress has been made since the discovery of the HGPS-causing mutation in 2003, and the relationship between the genotype and phenotype is quite well understood.^68−71^ Despite several years of research, the relationships between genotypes and phenotypes in laminopathies and in particular the HGPS remain poorly understood. Lamins A/C are expressed in many body tissues, even though modifications in lamins impact the overall mechanotransduction cell behavior; the outcome is especially manifest in tissues such as the heart and skeletal muscles that are exposed to long-lasting mechanical load. Consequently, the cell mechanical properties (elasticity, relaxation time) and adhesion properties have been considerably studied in the past several years since they are closely linked to many important biological cell behaviors, including adhesion, division, mobility, differentiation, and deformation.^72−74^ Furthermore, mechanical properties have been suggested as a marker to discriminate between healthy and pathological cells. Of particular interest in this study is the involvement of A-type lamins in anchoring protein complexes that form a bridge between the INM and the cellular cytoskeleton, probably not only softening the cytosol but also weakening the cell adhesion properties.^75^

In this study, we found that when comparing NRVM carrying the HGPS c.1824 C > T, p.Gly608Gly mutation with NT and WT LMNA controls, the HGPS cells present reduced work of adhesion, decreased relaxation time, and increased percent relaxation as assessed by stress relaxation experiments. Moreover, they have abnormal beating force and frequency, and altered and irregular calcium transients, suggesting an arrhythmic phenotype. Additionally, immunostaining for Cx43 showed that PGR cells had a decreased percentage of Cx43 localization, further supporting the arrhythmic phenotype. Our data are in line with the findings of Rivera-Torres et al.^76^ that HGPS patients and a Zmpste24–/– mouse model of HGPS reported cardiac electrical defects associated with the altered localization of Cx43 and abnormal repolarization and cardiomyocyte connectivity, all factors that may increase the risk of arrhythmia and premature death in patients at advanced disease stages. In another study by Prakash et al.,^77^ the authors found that diastolic dysfunction, appearing early in life with age-related decline, was the most prevalent cardiac abnormality in a small study of 27 patients with HGPS. Despite a high prevalence of diastolic dysfunction, no patients reported documented arrhythmias. However, the authors pointed out that further longitudinal research is still needed to determine whether some patients develop the symptom with further progression of the disease.

Our results indicate that NRVM carrying the progeria mutation presents an abnormal cardiomyocyte contraction associated with a decreased Cx43 localization, which correlates with the findings of Rivera-Torres et al.^76^ The abnormality in cardiomyocyte function can be attributed to the decreased cell adhesion found in the HGPS cells. As aforementioned, adhesion between cardiomyocytes is essential for the heart to function as an electromechanical syncytium;^78^ therefore, an alteration in cell adhesion can lead to an arrhythmogenic cardiomyocyte function, which is a phenotype presented in progeria patients with advanced disease stages. Our data also correlate with those of Macías^71^ et al. since they found both Cx43 mislocalization and alteration in heart rate variability. They also found tubulin-cytoskeleton disorganization, something we were not able to find probably because our cells were from NRVM while they were studying older mice.

Finally, although several studies have shown an increased Young’s modulus in fibroblasts carrying the progeria mutation, little has been reported about the stiffness of HGPS cardiomyocytes.^12,21,79^ In this study, we only observed a non-significant trend of a decrease in Young’s modulus of HGPS NRVM. Our results were correlated with the analysis of the actin filaments and microtubules in which no significant differences were observed between the groups regarding morphology and actin filament thickness. Interestingly, the HGPS NRVM model shows different biomechanics when compared to other laminopathies causing dilated cardiomyopathy (DCM) and limb-girdle muscular dystrophy. Indeed, in both the last models we observed a significant decrease in stiffness^25^ along with severe alterations in the actin filament network,^24^ suggesting that different LMNA mutations trigger distinct molecular mechanisms, ultimately leading to markedly different phenotypes.

### Study Limitations

Even though AFM can be used to evaluate the mechano-dynamic behavior of living cells, there are some parameters that can vary during the analysis, such as the fact that the orientation of actin-myosin filaments inside a cardiomyocyte is anisotropic, and thus, different portions of the cardiomyocytes may show different extents of movement and contractile forces with each beat. In this regard, rising the cell number analyzed and using an optical microscope to make the analysis can appreciably lessen external variabilities within the sample. Additionally, lateral modes of the contraction may not be accounted for by the simple z-movement of the AFM cantilever. However, in our study, all the physical and physiological cues were kept constant and therefore the calculated value can be used as a comparison between control and affected cells. Lastly, our AFM findings correlate with our biological analysis, and thus, our calculated beating force might be comparable for all specimens.

## Conclusions

Our study shows that the progeria c.1824 C > T (p.Gly608Gly) mutation caused a decrease in cell adhesion and relaxation time properties in cardiomyocytes that may lead to the impairment of force production. Furthermore, we found altered frequency and rhythm of spontaneous beating and calcium handling in a single-cardiomyocyte cell model, along with a decrease in Cx43 localization, suggesting that disrupted mechanotransduction and mechanosignaling lead to electrophysiological dysfunction in HGPS cardiomyocytes. These findings demonstrate how the cytoskeleton, sarcolemmal structures, and nucleoskeleton (LMNA) are all connected in the NRVM. Although the *in vitro* model presents limitations, it is intriguing that the changes found in cardiomyocytes recapitulate the clinical phenotype of progeria cardiomyopathy patients, who typically present mislocalization of Cx43. Cell indentation by atomic force microscopy represents a unique tool to complement cardiac cell biology investigations and elucidates the complex mechanisms leading to cardiac dysfunction in HGPS.

## Notes

Institutional Review Board Statement: “The study was conducted according to the guidelines of the Declaration of Helsinki and approved by the Institutional Review Board of the University of Colorado Denver (protocol code 00235 and date of approval July 30, 2019).
